# Evaluation of Conventional and Hyaluronic Acid-Coated Thymoquinone Liposomes in an In Vitro Model of Dry Eye

**DOI:** 10.3390/pharmaceutics15020578

**Published:** 2023-02-08

**Authors:** Elisa Landucci, Costanza Mazzantini, Maura Calvani, Domenico E. Pellegrini-Giampietro, Maria Camilla Bergonzi

**Affiliations:** 1 Department of Health Sciences, Section of Clinical Pharmacology and Oncology, University of Florence, Viale Pieraccini 6, 50139 Florence, Italy; 2 Division of Pediatric Oncology/Hematology, Meyer University Children’s Hospital, Viale Pieraccini 6, 50139 Florence, Italy; 3 Department of Chemistry, University of Florence, Via Ugo Schiff 6, Sesto Fiorentino, 50019 Florence, Italy

**Keywords:** dry eye, hyperosmolarity, thymoquinone, liposomes, hyaluronic acid, anti-inflammatory, antioxidants, mtROS

## Abstract

Dry eye disease (DED) is a common ocular disorder characterized by an inadequate lubrication of the eye by tears leading to inflammation and the alteration of the ocular surface. Current treatments are often limited due to their side effects and ineffectiveness. Thymoquinone (TQ) is a natural compound present in the essential oil of *Nigella sativa* L., with anti-inflammatory and antioxidant activities. In this study, conventional and hyaluronic acid-coated liposomes were developed to improve TQ activity at ocular level. In the present study, the cytoprotective effects of TQ or TQ liposomes were assessed against oxidative and inflammatory processes in human corneal epithelial cells (HCE-2). Hyperosmolarity conditions (450 mOsm) were used as a model of DED. Interleukin-1β (IL-1β), Interleukin-6 (IL-6) and tumor necrosis factor (TNFα) were quantified by quantitative real-time polymerase chain reaction (RT-qPCR); COX-2 and Phospho-NF-κB p65 (p-p65) by Western blotting (WB). Moreover, the mitochondrial reactive oxygen species (mtROS) levels were measured by MitoSOX assay. The hyperosmotic treatment induced a significant increase of the proinflammatory genes and proteins expression that were significantly decreased in the liposomes-treated cells. The coincubation with hyaluronic acid-coated liposomes significantly reverted the increase of mtROS production, evidently stimulated by the hyperosmotic stress. Our data suggest that TQ-loaded liposomes have potential as a therapeutic agent in dry eye disease, improving the TQ efficacy.

## 1. Introduction

Dry eye disease (DED) is a common ocular disorder affecting tens of millions of people, with prevalence in Asian compared to Western countries, indicating that the etiology of DED depends on cultural and racial factors [1]. The primary cause of DED is the decreased production of tears, such as the aqueous tear deficiency type of dry eye seen in Sjögren’s syndrome, but other conditions may also contribute to an unstable tear film [2]. There are many clinical conditions of patients with DED, hence the need to give a clinical definition that encompasses most of these. Tsubota and colleagues proposed a new definition: “Dry eye is a multifactorial disease characterized by a persistently unstable and/or deficient tear film causing discomfort and/or visual impairment, accompanied by variable degrees of ocular surface epitheliopathy, inflammation and neurosensory abnormalities” [3]. Tear film instability and hyperosmolarity induced ocular surface inflammation [4]. Macromolecular alterations and damage to ocular epithelial cells and lacrimal glands caused by oxidative stress induced an increase of inflammation condition [5]. Artificial tears are a common first-line therapy for DED; however, they are typically not sufficient or effective in managing the disease [6]. Different formulations, such as eye drops, topical lubricants, gels and ointments, are available [7]. In some patients there was no significative improvement with artificial tears and other formulations currently used in the clinic; hence, it is necessary to develop new formulations for dry eye. Current treatments are limited due to their side-effects and inefficacy. Natural compounds have a wide range of applications with deep-impact medical and healthcare fields, and numerous delivery systems, based on natural products, have been developed to optimize their biopharmaceutical properties. Thymoquinone (TQ) is the main constituent of the essential oil of *Nigella sativa* L. with high therapeutic potential [8] and it is known for its pharmacological properties as anti-inflammatory, antioxidant, antimicrobial, immunomodulatory, anticancer, hypoglycemic and antihypertensive [9,10]. The TQ efficacy in many neurological diseases, such as Parkinson’s and Alzheimer’s, was observed for its antioxidant, anti-inflammatory and apoptotic effects [11]. Furthermore, the neuroprotective effect of TQ was reported in a model of epilepsy in vitro [12]. The protective effect of TQ against H_2_O_2_-induced oxidative stress in human retinal pigment epithelium cells, has been evaluated [13]. TQ has anti-inflammatory properties in experimental DED model and decreased corneal neovascularization in a rat model [14]. Furthermore, TQ regulated the levels of pro- and anti-inflammatory mediators involved in corneal neovascularization [15], and significantly reduced the ocular symptoms in ovalbumin induced allergic conjunctivitis in BALB/c mice [16].

However, the use of TQ is very limited due to its low stability, solubility and bioavailability, as well as its sensitivity to light and pH, to prevent the reaching of the target sites [17]. In the last years, the limits of natural substances have been improved using nanotechnologies and drug delivery systems. Polymeric micelles improved the solubility and permeability of TQ, enhanced the TQ antimigration activity and inhibited human SH-SY5Y neuroblastoma cell migration [18]. As previously reported, liposomal formulations (LP-TQ) and, in particular, hyaluronic acid-coated liposomes (LP-TQ-HA), reduced the toxicity showed by TQ at high doses in HCE-2 and HConEC cells. Liposomes improved the absorption at the nucleus level, with a more pronounced effect for HA-coated liposomes [19].

Hyaluronic acid (HA) is a natural macromolecule present in the vitreous body of the eye; it is used as a thickener and for its physical properties comparable to tear glycoproteins, which can coat the corneal epithelium. HA may play a role in inflammation and wound healing and it exerts long-term beneficial effects on corneal epithelium regeneration [20]. Diluted solutions of HA have been employed as artificial tears to treat DED symptoms as it improves the hydration of the ocular surface and reduces friction [21]. Furthermore, HA is a mucoadhesive polymer and its interactions with the mucus layer or eye tissues increases the persistence of the drug in the precorneal region [22]. In addition, HA binds the CD44 receptors localized in the ophthalmic cells, particularly in human conjunctiva and cornea epithelial cells, and in the retinal pigment epithelium [23].

Liposomes are biocompatible and biodegradable nanovesicles applied to improve the bioavailability of ophthalmic drugs after topical instillation [24]. The main constituents of liposomes are phospholipids that are also components of the tear film lipid layer. Furthermore, liposomes reduce aqueous evaporation increasing tear film stability [24]. To enhance adherence to the eye surface, the dispersion of the liposomes in mucoadhesive gels [25] or the coating with mucoadhesive polymers, such as HA and chitosan [26], was proposed. HA coating realizes steric stabilization and active targeting and improves liposomal stability [19,27].

In the present study, for the first time, TQ-loaded liposomes (LP-TQ) and hyaluronic acid-coated TQ liposomes (LP-TQ-HA) show their cytoprotective effects against oxidative and inflammatory processes in human corneal epithelial cells (HCE-2) in a model of DED in vitro. Hyperosmolarity conditions (450 mOsm) were used as a model of DED [28]. The effect of the formulations was compared with free TQ. Both liposomes are consisted of phosphatidylcholine and Plurol Oleique, a liquid lipid used to improve the loading capacity, and one formulation was coated with 0.1% *w*/*v* HA. The liposomes were developed to increase TQ solubility and availability at ocular level [19]. Interleukin-1β (IL-1β), Interleukin-6 (IL-6) and tumor necrosis factor (TNFα) were quantified by quantitative real-time polymerase chain reaction (RT-qPCR) and IL-1β, COX-2 and Phospho-NF-κB p65 (p-p65) by Western blotting (WB). The mitochondrial reactive oxygen species (mtROS) levels were measured by MitoSOX assay.

## 2. Materials and Methods

### 2.1. Materials

The TQ, mucin from porcine stomach type II, were purchased from Sigma-Aldrich (Milan, Italy). Plurol Oleique CC 497 was supplied by Gattefossè sas (Saint-Priest, France). Phospholipid Gmbh (Cologne, Germany) supplied egg phosphatidylcholine and Phospholipon 90 G. Sodium hyaluronate (M.W. 1000 KDa, HA) was obtained from Altergon, City, Avellino, Italy. The water was from the Milli-Qplus system from Millipore (Milford, CT, USA).

### 2.2. Preparation of Liposomal Formulations

The TQ-loaded liposomes (LP-TQ) and HA-coated TQ liposomes (LP-TQ-HA), were prepared according to the literature [19]. The TQ, egg phosphatidylcholine and Plurol Oleique were dissolved in dichloromethane. The solvent was evaporated and the dry lipid film was hydrated with deionized water. The dispersion was shaken for 30 min in a water bath at 50 °C and then sonicated with an ultrasonic probe for 2 min and 30 s. The HA coating was achieved by the drop-wise method [29], adding 2 mL of 0.1% *w*/*v* solution of HA in deionized water to 2 mL of LP-TQ dispersion. The LP-TQ-HA has a final concentration of 0.5 mg/mL of TQ [19].

### 2.3. Characterization of Liposomes

Hydrodynamic diameter, polydispersity index (PdI), and zeta-potential were determined by Dinamic Light Scattering (DLS) and Electrophoretic Light Scattering (ELS), using a Zsizer Nano series ZS90 (Malvern Instruments, Malvern, UK). The encapsulation efficiency of LP-TQ and LP-TQ-HA was determined by the dialysis bag method, as previously reported [19].

### 2.4. Mucoadhesion Study

The mucoadhesion interaction was determined by the zeta-potential measurements of the mucin solutions and the mixtures of mucin and formulations using a Zsizer Nano series ZS90 (Malvern Instruments, Malvern, UK) [30,31]. In total, 1 mL of LP-TQ or LP-TQ-HA formulations was mixed with 1 mL of mucin suspension at different percentages (0.5%, 1%, 2% and 3%) and incubated in the ultrasound bath for 15 min. The samples were subsequently diluted with deionized water and analyzed at the ELS for the measurement of the zeta-potential.

### 2.5. Human Corneal Epithelial Cells (HCE-2)

As previously reported, the human corneal epithelial cells (HCE-2) were incubated in serum-free medium supplemented with bovine pituitary extract (BPE), epidermal growth factor, hydrocortisone and insulin [32], then they were plated on coated flasks and incubated in a humidified incubator at 37 °C. The medium was changed twice a week. The cells were split in new flasks after reaching the confluence.

### 2.6. Analysis of In Vitro Cytotoxicity

The HCE-2 cells were plated in 24-well plates and, after they had reached the confluence, were exposed to the drugs treatment, suitably diluted. The medium was used as positive control and BAK 0.01% was used as negative control for maximum cell death. Cells were incubated with TQ, LP-TQ or LP-TQ-HA for 5 h.

#### 2.6.1. MTT Assay

The viability of corneal epithelial cells exposed to TQ-free or formulated (5 µM) for 5 h was evaluated by MTT assay. From each well a part of the medium was withdrawn for LDH assay. Then, the MTT (1 mg/mL) was added to the cells [19]. After removing the MTT-containing solution, dimethyl sulfoxide (DMSO) was added to the wells to dissolve the formazan crystal formations, and the absorbance of MTT was read at 550 and 690 nm. The medium was used as a positive control. Cell viability was expressed as a percentage of cells incubated only in the vehicle at the corresponding exposure time.

#### 2.6.2. LDH Assay

Damage in human corneal cells was quantitatively assessed by measuring the amount of LDH released by the damaged cells into the extracellular fluid, 5 h after drug exposure, by LDH kit, as previously described [19]. The LDH level corresponding to complete cell death was determined for each experiment by analyzing sister cultures exposed to 0.01% BAK. Background LDH release was determined in drug-unexposed control cultures and subtracted from all experimental values.

### 2.7. HCE-2 Exposure to Hyperosmolarity

HCE-2 cells were suspended and plated in pretreated 24-well plates (approximately 4 × 104 cells/cm^2^). Once they reached about 70–80% of confluence, the medium was removed and the cells were exposed for 5 h with hyperosmotic (450 mOsM) medium, obtained by sodium chloride (NaCl, 69 mM) addition [28]. The medium’s osmolarity was measured by the Advanced**^®^** Model 3320 Micro-Osmometer (Advanced Instruments, Norwood, OH, USA). Hyperosmolar cells were treated with TQ- (5 µM) free or formulated. Gene expression, protein levels and MitoSOX assay were performed after the treatments.

### 2.8. Evaluation of Interleukin-1b (IL-1b), Interleukin-6 (IL-6) and Tumor Necrosis Factor α (TNFα) Gene Expression by Quantitative Real-Time Polymerase Chain Reaction (RT-qPCR)

Total RNA was isolated from HCE-2 cells using Trizol Reagent (Life Technologies). One µg of RNA was retrotranscribed using iScript (Bio-Rad, Milan, Italy). RT-PCR was performed as reported [33]. The following primers were used: IL-1β human: forward 5′-CAGCTACGAATCTCCGACCACCAC-3′ and reverse 5′-GCCTCGTTATCCCATGTGTCGAAG-3′; IL-6 human: forward 5′-AGCAGCAAAGAGGCACTGGCAG-3′ and reverse 5′-ATCTGCACAGCTCTGGCTTGTTCC-3′; TNF-α human: forward 5′-ACCAAGGTCAACCTCCTCTCTGCC-3′ and reverse 5′-CCAAAGTAGACCTGCCCAGACTCG-3′; 18S human: forward 5′-CGGCTACCACATCCAAGGAA-3′ and reverse 5′-GCTGGAATTACCGCGGCT-3′.

### 2.9. Evaluation of Interleukin-1β (IL-1β), Cyclooxygenase-2 (COX-2) and Phosphor Nuclear Factor NF-Kappa-B p65 Subunit (p-p65) Protein Expression by Western Blotting (WB)

HCE-2 cells were washed with cold 0.01 M PBS, pH 7.4 at the end of the treatment and dissolved in 1% SDS. The BCA (bicinchoninic acid) protein assay was used to quantify the total protein levels. Lysates (20 μg/lane of protein) were resolved by electrophoresis on a 4–20% SDS-polyacrylamide gel (Bio-Rad Laboratories, Hercules, CA, USA) and transferred onto nitrocellulose membranes. Blots were blocked for 1 h at room temperature in 20 mM Tris-buffered saline, pH 7.6, 0.1% Tween 20 (TBS-T) containing 5% non-fat dry milk, and then incubated overnight at 4 °C with rabbit polyclonal antibodies against IL-1β and COX-2 (all from Abcam, Cambridge CB2 0AX, UK), monoclonal rabbit antibodies against Phospho-NF-κB p65 (Ser536) (93H1) (Cell Signaling Technology, Beverly, MA, USA), diluted 1:1000 in TBS-T containing 5% bovine serum albumin. As loading control, the monoclonal anti-β-actin antibody was from Sigma (St. Louis, MO, USA). Immunodetection was performed with secondary antibodies (1:3000 anti-mouse or antirabbit IgG from donkey, Amersham Biosciences, Amersham, UK) conjugated to horseradish peroxidase in TBS-T containing 5% non-fat dry milk. Membranes were washed with TBS-T and then reactive bands were detected using chemiluminescence (ECLplus; Euroclone, Padova, Italy). Quantitative analysis was performed using the QuantityOne analysis software (Bio-Rad, Hercules, CA, USA).

### 2.10. Evaluation of Mitochondrial Reactive Oxygen Species (mtROS)

As previously reported, the HCE-2 cells were stained with MitoSOX at the final concentration of 2.5 µM for 15 min, then washed with PBS, detached with accutase, and resuspended with PBS [34]. The stained cells were acquired using a MACSQuant Analyzer 10 Flow Cytometer (Miltenyi Biotec**^®^**, Bergisch Gladbach, Germany), and the data were analyzed by Flowlogic (Miltenyi Biotec**^®^**).

### 2.11. Statistical Analysis

Experiments were repeated ***n*** times and results expressed as a mean ± SEM. The statistical significance of HCE-2 cell viability was analyzed by one-way ANOVA with a post-hoc Dunnett and gene expression; protein levels and mtROS were analyzed by one-way ANOVA followed by the post-hoc Tukey’s w-test for multiple comparisons. All statistical analyses were performed by the GRAPH-PAD PRISM v. 8 for Windows (GraphPad Software, San Diego, CA, USA). A probability value (*p*) of < 0.05 was considered significant.

## 3. Results and Discussion

### Characterization of Liposomes

The physical and chemical parameters of LP-TQ and LP-TQ-HA were reported in Table 1 [19]. The HA coating was obtained using the drop by drop coating method [29], which is based on the hydrophobic interactions and hydrogen bonds between PC and HA [35,36,37,38]. The increase of the particle sizes from 146 ± 2 nm to 166 ± 3 nm and the change of zeta potential from −26 ± 3 to −36 ± 1 mV confirmed the HA deposition. TEM analyses also demonstrated this deposition around the liposomes [19]. The high value of potential for both LP-TQ and LP-TQ-HA contributes to increase the stability of the dispersion, reducing the aggregation phenomena.

The study of the mucoadhesion is of great importance to understand the efficacy of the formulation as a potential candidate for DED treatment. Different in vitro methods were reported [39,40,41]. In this study, zeta-potential determination was applied to study the mucoadhesive properties of LP-TQ-HA [30]. This is a common approach used for the investigation of mucoadhesive characteristics of biopolymers [42,43]. The mucoadhesive properties of the liposomes were evaluated by monitoring changes in the potential following incubation of the liposomal formulation with mucin [44]. The mucin has negative charge due to the carboxyl and sulphate groups of the oligosaccharide chains. The obtained value was around -10 mV, similar to the value reported in the literature [30,45,46]. The surface property of the mucin might be changed by the adhesion of the polymer, if the polymer is mucoadhesive [31]. The zeta-potential showed only a moderate shift when LP-TQ was added to the solutions of mucin (0.5%, 1%, 2% and 3% *w*/*v*), while in the presence of LP-TQ-HA a more pronounced change is observed (Table 2). The addition of the mucin produces a more negative potential in respect to mucin alone, according to the literature [30]. Furthermore, as the percentage of mucin increases, the interaction with the HA-coated formulation increases, and the potential approaches that of the mucin [30,47]. The DLS analyses also evidenced the increase of the sizes of HA-LP-TQ in the presence of mucin, confirming this interaction.

Previously, authors reported that both the formulations reduced the possible toxicity of a high dosage (30 and 60 µM) of TQ exposure in cornea and conjunctiva cells [19].

**Figure 1:**
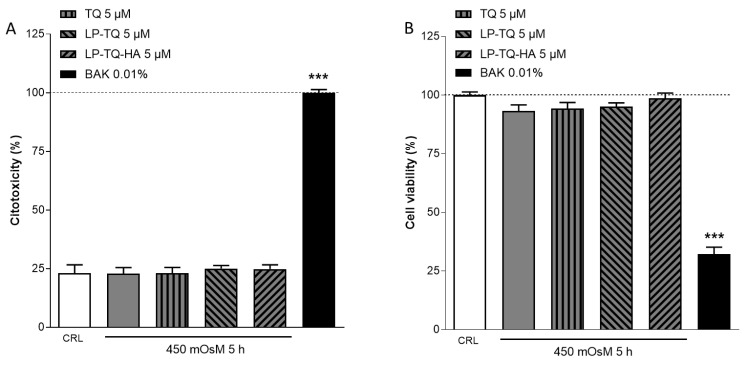
The evaluation of LDH and MTT assay in the HCE-2 cells after 5 h of incubation with hyperosmolarity media alone, or in the presence of TQ, LP-TQ or LP-TQ-HA. Data are expressed as percentages of the maximum degree of cell death BAK 0.01% (**A**) and as percentages of maximum cell viability (medium) (**B**); they represent the mean ± SEM of the mean of at least three experiments performed in quadruplicate. *** *p* < 0.001 vs. CRL (one-way ANOVA plus Dunnett’s).

Many studies suggest a crucial role of inflammatory and oxidative processes in the pathogenesis of DED and, in particular, tear film hyperosmolarity is accepted as a key pathogenic step, since it causes damage to the surface epithelium activating a cascade of inflammatory events at the ocular surface [48,49]. In this study, we analyzed the effect of the hyperosmolarity treatment alone or in presence of TQ and TQ-loaded liposomes at a lower concentration (5 µM) for a long time (5 h) on the cornea cell viability using LDH and MTT assays. As shown in Figure 1, the hyperosmolarity treatment did not induce cell toxicity in both conditions.

**Figure 2:**
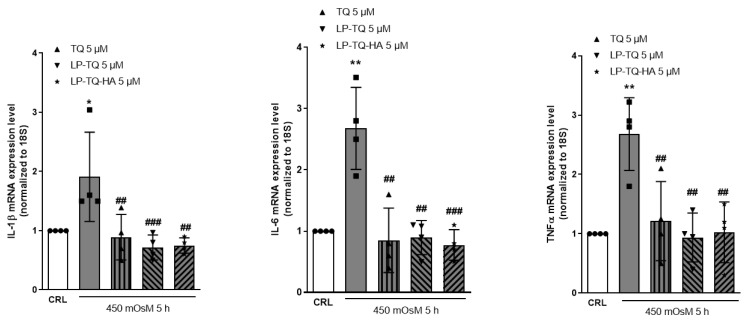
The mRNA levels of IL-1β, Il-6 and TNFα in HCE-2 cells exposed to hyperosmotic media and treated with TQ, LP-TQ and PL-TQ-HA. Dot blots show the results of four experiments. * *p* < 0.05 and ** *p* < 0.01 vs. CRL; ## *p* < 0.01 and ### *p* < 0.01 vs. hyperosmolarity (ANOVA + Tukey’s w-test).

The key role of proinflammatory cytokines in the tears of patients with DED, and other ocular surface diseases, was recently suggested by studies in literature [50]. A recent systematic review revealed that DED patients had higher tear levels of IL-1β, IL-6 and TNF-α, as compared to controls [51]. Based on these considerations, we analyzed the anti-inflammatory effect of TQ, LP-TQ and LP-TQ-HA under hyperosmolarity in HCE-2 cells. Figure 2 shows the mRNA levels of IL-1β, Il-6 and TNFα in corneal cells exposed to hyperosmotic medium. The treatment significantly increased the mRNA expression of the IL-1β, Il-6 and TNFα compared to normal control cells, as previously observed in in vitro and in vivo models [28,52]. However, the expression of these proinflammatory cytokines significantly decreased in the TQ and liposomes-treated cells (Figure 2).

**Figure 3:**
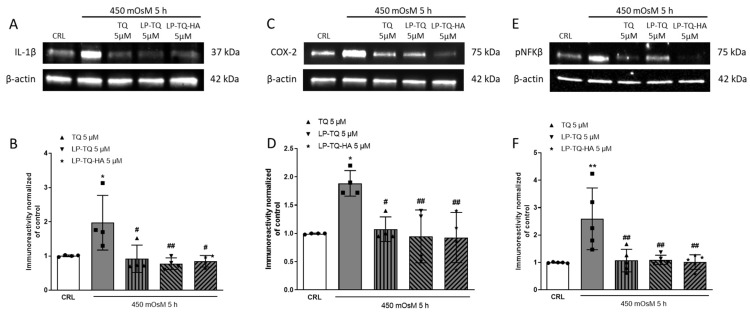
The effects of TQ on proinflammatory signaling in HCE-2 cells exposed to hyperosmotic media. Cells were exposed to 450 mOsM for 5 h and then processed for WB. The TQ-free and formulated were present in the incubation medium during hyperosmolarity exposure. (**A**,**C**,**E**) Representative blots for IL-1β or COX-2 or NF-kappa-B p65 or β-actin. (**B**,**D**,**F**) the quantitative analysis of immunoreactive bands, showing that TQ is able to reduce the increase levels of proinflammatory protein induced by 450 mOsM for 5 h. Dot blots show the results of four experiments. * *p* < 0.05 and ** *p* < 0.01 vs. CRL; # *p* < 0.05 and ## *p* < 0.01 vs. hyperosmolarity. (ANOVA + Tukey’s w-test).

Given the modifications observed at the gene level, we investigated whether these translated into protein modifications by analyzing some proinflammatory proteins, such as Il-1β, COX-2 and NF-kappa-B p65. In this model of DED in vitro, the increased level of this proinflammatory protein was reverted by the treatment with TQ, LP-TQ and LP-TQ-HA (Figure 3).

Many studies suggested a key role of inflammatory and oxidative processes in the pathogenesis of DED [49,50,53]. In particular, the mitochondrial function was related to the progression of DED as well as the outcome of this disease to the modulation of mitochondrial homeostasis [54]. DED patients had high levels of reactive oxygen species (ROS) and increased inflammatory markers in the tear film [55]. The oxidized protein levels were increased due to the high inflammatory activity in DED [5]. Key events in the pathogenesis of this disease were the tear film instability, its hyperosmolarity, the ocular surface damage and inflammation [49]. Hyperosmolarity caused damage to the epithelium surface by activating the cascade of inflammatory events in the ocular surface and, moreover, it induced a release of inflammatory mediators into the tears [56]. In the form of hyperosmolar culture medium, hyperosmolarity also induced oxidative stress in cultured primary HCE-2 cells. Additionally, oxidative stress affected corneal epithelial cells directly, causing irreversible macromolecular alterations and oxidative modifications of nuclear acids, lipids and proteins, and indirectly, via the increased expression of proinflammatory cytokines [28,57].

The prolonged local inflammation induced by oxidative stress was often the cause of the corneal injury [58]. An imbalance between antioxidant defenses and ROS, produced in large part by mitochondria, induces oxidative stress [59]. Chen and colleagues in 2022 demonstrated an improvement in the effects of DED reversing hyperosmolarity-mediated mitochondrial dysfunction in HCE-2 [60]. For this reason, we focused our attention on mtROS levels.

**Figure 4:**
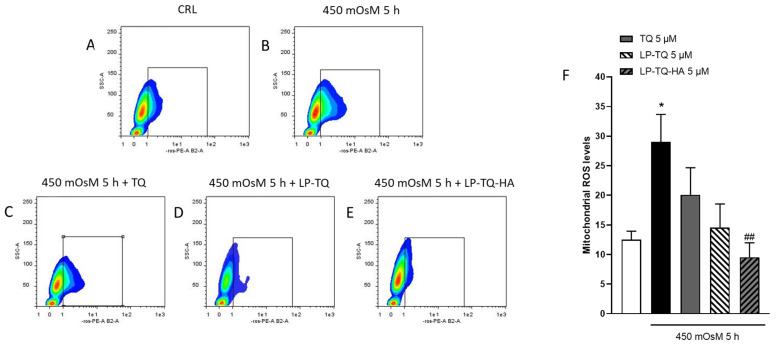
The effects of TQ on mtROS production in HCE-2 cells exposed to hyperosmotic media. Cells were exposed to 450 mOsM for 5 h and then processed for the quantification of mtROS by cytofluorimetric analysis. Free- and formulated TQ were present in the incubation medium during hyperosmolarity exposure. (**A**–**E**) representative plots showing the amount of ROS produced during the treatments. The red color represents the highest density within a population. With decreasing density, the color transitions from yellow over green to blue. (**F**) quantitative analysis of plots, showing that only LP-TQ-HA can reduce significantly the increase levels of mtROS induced by 450 mOsM for 5 h. Dot show the results of six experiments. * *p* < 0.05 vs. CRL; ## *p* < 0.01 vs. hyperosmolarity. (ANOVA + Tukey’s w-test).

In this study, oxidative stress was determined by the quantification of mtROS levels in HCE-2 cells through the MitoSOX™ Red mitochondrial assay that detected the superoxide in the mitochondria of live cells. The measurement of mtROS levels revealed that hyperosmotic stress markedly stimulated ROS production, and this effect was reverted by the coincubation with TQ, LP-TQ and, significantly, with LP-TQ-HA at the concentration of 5 µM for 5 h, as shown in Figure 4. As previously reported, LP-TQ-HA reduced the toxicity that TQ showed at high doses in HCE-2 cells and improved the absorption at the nucleus level with a more pronounced effect for HA-coated liposomes [19]. All these results confirm that these liposomes could represent a valid therapeutic agent against DED.

Many studies reported that TQ has potential protective effects on cardiovascular disorder and mitochondrial protection, acting against oxidative stress and organelle damages [61,62]. Moreover, the antioxidant and anti-inflammatory effects of TQ have been clearly proven in many other diseases [63]. Liang and colleagues showed that TQ played an inhibitory role in UVA-induced oxidative stress, inflammation and the mitochondrial apoptosis of human skin keratinocytes [64]. The antioxidant properties of HA are well known, as reported by Almalik and co-workers in 2018, where it was shown that HA is involved in inflammatory response, due to its antioxidant scavenging activity [65,66]. According to these data, in our work, HA-coated liposomes significantly reduced the mtROS levels induced by 450 mOsM for 5 h, and this could be explained by a possible synergistic effect of TQ with HA. Furthermore, HA can also increase the adhesion to the cell due to its mucoadhesive properties [67]; in addition, it could also act as scavenger, since it is a negative-charged polysaccharide with a reductive end [68,69].

## 4. Conclusions

In the present investigation, liposomes were used as carriers to improve TQ solubility and availability at the ocular level. It investigated the efficacy of TQ liposomal formulations in a model of DED in vitro. The liposomes consist of phosphatidylcholine and Plurol Oleique, and one formulation has a HA coating that provides the mucoadhesive properties to formulation. Moreover, the antioxidative and anti-inflammatory functions of TQ were used to achieve the goal in DED. The hyperosmotic treatment induced a significant increase of the proinflammatory genes and proteins expression that was significantly decreased in the cells treated with TQ, and this effect was maintained by the liposomes. The hyperosmotic stress markedly stimulated mtROS production and the coincubation with LP-TQ-HA significantly reverted this increase, highlighting the mucoadhesive and antioxidant effect of HA. These findings suggest that these liposomes may potentially be applied as a therapeutic agent against DED, after further studies in model in vivo that could better demonstrate the benefit of the HA coating.

## Figures and Tables

**Table 1:** Physical and chemical characterization of TQ-loaded conventional liposomes (LP-TQ), and HA-coated TQ liposomes (LP-TQ-HA) (mean ± SEM, *n* = 3).

Sample	Size (nm)	PdI	Zeta-Potential (mV)	EE%
LP-TQ	146 ± 0.17	0.15 ± 0.08	−26 ± 0.59	73 ± 0.35
LP-TQ-HA	166 ± 0.23	0.27 ± 0.02	−36 ± 0.17	73 ± 0.47

**Table 2:** Zeta-potential (mV) of different solutions of mucin and mixtures with LP-TQ or LP-TQ-HA.

Sample	Zeta-Potential
Mucin 0.5%	−13.08 ± 0.38
Mucin 0.5% + LP-TQ	−24.52 ± 1.0
Mucin 0.5% + HA-LP-TQ	−22.15 ± 0.96
Mucin 1%	−11.12 ± 0.27
Mucin 1% + LP-TQ	−23.20 ± 0.93
Mucin 1% + HA-LP-TQ	−18.05 ± 1.40
Mucin 2%	−11.41 ± 1.25
Mucin 2% + LP-TQ	−22.90 ± 0.76
Mucin 2% + HA-LP-TQ	−14.09 ± 0.93
Mucin 3%	−9.23 ± 0.73
Mucin 3% + LP-TQ	−21.09 ± 0.84
Mucin 3% + HA-LP-TQ	−12.25 ± 1.26

## Data Availability

The data presented in this study are available on request from the corresponding authors.

## References

[1] Uchino M., Nishiwaki Y., Michikawa T., Shirakawa K., Kuwahara E., Yamada M., Dogru M., Schaumberg D.A., Kawakita T., Takebayashi T. (2011). Prevalence and risk factors of dry eye disease in Japan: Koumi study. Ophthalmology.

[2] Tsubota K., Yokoi N., Shimazaki J., Watanabe H., Dogru M., Yamada M., Kinoshita S., Kim H.M., Tchah H.W., Hyon J.Y. (2017). New Perspectives on Dry Eye Definition and Diagnosis: A Consensus Report by the Asia Dry Eye Society. Ocul. Surf..

[3] Tsubota K., Pflugfelder S.C., Liu Z., Baudouin C., Kim H.M., Messmer E.M., Kruse F., Liang L., Carreno-Galeano J.T., Rolando M. (2020). Defining Dry Eye from a Clinical Perspective. Int. J. Mol. Sci..

[4] Stern M.E., Gao J., Siemasko K.F., Beuerman R.W., Pflugfelder S.C. (2004). The role of the lacrimal functional unit in the pathophysiology of dry eye. Exp. Eye Res..

[5] Dogru M., Kojima T., Simsek C., Tsubotav K. (2018). Potential Role of Oxidative Stress in Ocular Surface Inflammation and Dry Eye Disease. Investig. Ophthalmol. Vis. Sci..

[6] Jones L., Downie L.E., Korb D., Benitez-del-Castillo J.M., Dana R., Deng S.X., Dong P.N., Geerling G., Hida R.Y., Liu Y. (2017). TFOS DEWS II Management and Therapy Report. Ocul. Surf..

[7] Ling J., Chan B.C.-L., Tsang M.S.-M., Gao X., Leung P.C., Lam C.W.-K., Hu J.-M., Wong C.K. (2021). Current Advances in Mechanisms and Treatment of Dry Eye Disease: Toward Anti-inflammatory and Immunomodulatory Therapy and Traditional Chinese Medicine. Front. Med..

[8] Malik S., Singh A., Negi P., Kapoor V.K. (2021). Thymoquinone: A small molecule from nature with high therapeutic potential. Drug Discov. Today.

[9] Banerjee S., Padhye S., Azmi A., Wang Z., Philip P.A., Kucuk O., Sarkar F.H., Mohammad R.M. (2010). Review on molecular and therapeutic potential of thymoquinone in cancer. Nutr. Cancer.

[10] Darakhshan S., Bidmeshki Pour A., Hosseinzadeh Colagar A., Sisakhtnezhad S. (2015). Thymoquinone and its therapeutic potentials. Pharmacol. Res..

[11] Farkhondeh T., Samarghandian S., Shahri A.M.P., Samini F. (2018). The Neuroprotective Effects of Thymoquinone: A Review. Dose-Response.

[12] Landucci E., Mazzantini C., Buonvicino D., Pellegrini-Giampietro D.E., Bergonzi M.C. (2021). Neuroprotective Effects of Thymoquinone by the Modulation of ER Stress and Apoptotic Pathway in In Vitro Model of Excitotoxicity. Molecules.

[13] Hu X., Liang Y., Zhao B., Wang Y. (2019). Thymoquinone protects human retinal pigment epithelial cells against hydrogen peroxide induced oxidative stress and apoptosis. J. Cell. Biochem..

[14] Kocatürk T., Erkan E., Meteoğlu İ., Ekici M., Büyüköztürk A.K., Yavaşoğlu İ., Çakmak H., Dayanır V., Balkaya M. (2018). Effects of Topical Thymoquinone in an Experimental Dry Eye Model. Turkish J. Ophthalmol..

[15] Salem M.L. (2005). Immunomodulatory and therapeutic properties of the Nigella sativa L. seed. Int. Immunopharmacol..

[16] Hayat K., Asim M.R., Nawaz M., Li M., Zhang L., Sun N. (2011). Ameliorative effect of thymoquinone on ovalbumin-induced allergic conjunctivitis in Balb/c mice. Curr. Eye Res..

[17] Salmani J.M.M., Asghar S., Lv H., Zhou J. (2014). Aqueous solubility and degradation kinetics of the phytochemical anticancer thymoquinone; probing the effects of solvents, pH and light. Molecules.

[18] Bergonzi M.C., Vasarri M., Marroncini G., Barletta E., Degl’Innocenti D. (2020). Thymoquinone-Loaded Soluplus^®^-Solutol^®^ HS15 Mixed Micelles: Preparation, In Vitro Characterization, and Effect on the SH-SY5Y Cell Migration. Molecules.

[19] Landucci E., Bonomolo F., De Stefani C., Mazzantini C., Pellegrini-Giampietro D.E., Bilia A.R., Bergonzi M.C. (2021). Preparation of Liposomal Formulations for Ocular Delivery of Thymoquinone: In Vitro Evaluation in HCEC-2 e HConEC Cells. Pharmaceutics.

[20] Litwiniuk M., Krejner A., Grzela T. (2016). Hyaluronic Acid in Inflammation and Tissue Regeneration. Wounds Compend. Clin. Res. Pract..

[21] Sánchez-González J.M., De-Hita-Cantalejo C., SánchezGonzález M.C. (2020). Crosslinked hyaluronic acid with liposomes and crocin for management symptoms of dry eye disease caused by moderate meibomian gland dysfunction. Int. J. Ophthalmol..

[22] Ludwig A. (2005). The use of mucoadhesive polymers in ocular drug delivery. Adv. Drug Deliv. Rev..

[23] García-Posadas L., Contreras-Ruiz L., López-García A., Villarón Álvarez S., Maldonado M.J., Diebold Y. (2012). Hyaluronan receptors in the human ocular surface: A descriptive and comparative study of RHAMM and CD44 in tissues, cell lines and freshly collected samples. Histochem. Cell Biol..

[24] Mishra G.P., Bagui M., Tamboli V., Mitra A.K. (2011). Recent applications of liposomes in ophthalmic drug delivery. J. Drug Deliv..

[25] Fahmy A.M., Hassan M., El-Setouhy D.A., Tayel S.A., Al-Mahallawi A.M. (2021). Statistical optimization of hyaluronic acid enriched ultradeformable elastosomes for ocular delivery of voriconazole via Box-Behnken design: In vitro characterization and in vivo evaluation. Drug Deliv..

[26] Kari O.K., Tavakoli S., Parkkila P., Baan S., Savolainen R., Ruoslahti T., Johansson N.G., Ndika J., Alenius H., Viitala T. (2020). Light-Activated Liposomes Coated with Hyaluronic Acid as a Potential Drug Delivery System. Pharmaceutics.

[27] Peer D., Florentin A., Margalit R. (2003). Hyaluronan is a key component in cryoprotection and formulation of targeted unilamellar liposomes. Biochim. Biophys. Acta Biomembr..

[28] Ali S., Davinelli S., Mencucci R., Fusi F., Scuderi G., Costagliola C., Scapagnini G. (2021). Crosslinked Hyaluronic Acid with Liposomes and Crocin Confers Cytoprotection in an Experimental Model of Dry Eye. Molecules.

[29] Mady M.M., Darwish M.M., Khalil S., Khalil W.M. (2009). Biophysical studies on chitosan-coated liposomes. Eur. Biophys. J..

[30] Graça A., Gonçalves L., Raposo S., Ribeiro H., Marto J. (2018). Useful In Vitro Techniques to Evaluate the Mucoadhesive Properties of Hyaluronic Acid-Based Ocular Delivery Systems. Pharmaceutics.

[31] Takeuchi H., Thongborisute J., Matsui Y., Sugihara H., Yamamoto H., Kawashima Y. (2005). Novel mucoadhesion tests for polymers and polymer-coated particles to design optimal mucoadhesive drug delivery systems. Adv. Drug Deliv. Rev..

[32] Mencucci R., Favuzza E., Bottino P., Mazzantini C., Zanotto E., Pellegrini-Giampietro D.E., Landucci E. (2020). A new ophthalmic formulation containing antiseptics and dexpanthenol: In vitro antimicrobial activity and effects on corneal and conjunctival epithelial cells. Exp. Eye Res..

[33] Buonvicino D., Ranieri G., Pratesi S., Guasti D., Chiarugi A. (2019). Neuroimmunological characterization of a mouse model of primary progressive experimental autoimmune encephalomyelitis and effects of immunosuppressive or neuroprotective strategies on disease evolution. Exp. Neurol..

[34] Calvani M., Cavallini L., Tondo A., Spinelli V., Ricci L., Pasha A., Bruno G., Buonvicino D., Bigagli E., Vignoli M. (2018). β 3-Adrenoreceptors Control Mitochondrial Dormancy in Melanoma and Embryonic Stem Cells. Oxid. Med. Cell. Longev..

[35] Zeng W., Li Q., Wan T., Liu C., Pan W., Wu Z., Zhang G., Pan J., Qin M., Lin Y. (2016). Hyaluronic acid-coated niosomes facilitate tacrolimus ocular delivery: Mucoadhesion, precorneal retention, aqueous humor pharmacokinetics, and transcorneal permeability. Colloids Surf. B Biointerfaces.

[36] Gómez Gaete C., Tsapis N., Silva L., Bourgaux C., Fattal E. (2008). Morphology, structure and supramolecular organization of hybrid 1,2-dipalmitoyl-sn-glycero-3-phosphatidylcholine-hyaluronic acid microparticles prepared by spray drying. Eur. J. Pharm. Sci..

[37] Mayol L., Quaglia F., Borzacchiello A., Ambrosio L., Rotonda M.I.L. (2008). A novel poloxamers/hyaluronic acid in situ forming hydrogel for drug delivery: Rheological, mucoadhesive and in vitro release properties. Eur. J. Pharm. Biopharm..

[38] El Kechai N., Mamelle E., Nguyen Y., Huang N., Nicolas V., Chaminade P., Yen-Nicolaÿ S., Gueutin C., Granger B., Ferrary E. (2016). Hyaluronic acid liposomal gel sustains delivery of a corticoid to the inner ear. J. Control. Release.

[39] Salzillo R., Schiraldi C., Corsuto L., D’Agostino A., Filosa R., De Rosa M., La Gatta A. (2016). Optimization of hyaluronan-based eye drop formulations. Carbohydr. Polym..

[40] Menchicchi B., Fuenzalida J.P., Hensel A., Swamy M.J., David L., Rochas C., Goycoolea F.M. (2015). Biophysical analysis of the molecular interactions between polysaccharides and mucin. Biomacromolecules.

[41] Ivarsson D., Wahlgren M. (2012). Comparison of in vitro methods of measuring mucoadhesion: Ellipsometry, tensile strength and rheological measurements. Colloids Surf. B Biointerfaces.

[42] Mendes A., Sevilla Moreno J., Hanif M., EL Douglas T., Chen M., Chronakis I. (2018). Morphological, Mechanical and Mucoadhesive Properties of Electrospun Chitosan/Phospholipid Hybrid Nanofibers. Int. J. Mol. Sci..

[43] Nikogeorgos N., Efler P., Kayitmazer A.B., Lee S. (2015). “Bio-glues” to enhance slipperiness of mucins: Improved lubricity and wear resistance of porcine gastric mucin (PGM) layers assisted by mucoadhesion with chitosan. Soft Matter.

[44] Bhatta R.S., Chandasana H., Chhonker Y.S., Rathi C., Kumar D., Mitra K., Shukla P.K. (2012). Mucoadhesive nanoparticles for prolonged ocular delivery of natamycin: In vitro and pharmacokinetics studies. Int. J. Pharm..

[45] Fathalla Z.M.A., Khaled K.A., Hussein A.K., Alany R.G., Vangala A. (2016). Formulation and corneal permeation of ketorolac tromethamine-loaded chitosan nanoparticles. Drug Dev. Ind. Pharm..

[46] Menchicchi B., Fuenzalida J.P., Bobbili K.B., Hensel A., Swamy M.J., Goycoolea F.M. (2014). Structure of chitosan determines its interactions with mucin. Biomacromolecules.

[47] Aguilera-Garrido A., Molina-Bolívar J.A., Gálvez-Ruiz M.J., Galisteo-González F. (2019). Mucoadhesive properties of liquid lipid nanocapsules enhanced by hyaluronic acid. J. Mol. Liq..

[48] Baudouin C., Aragona P., Messmer E.M., Tomlinson A., Calonge M., Boboridis K.G., Akova Y.A., Geerling G., Labetoulle M., Rolando M. (2013). Role of hyperosmolarity in the pathogenesis and management of dry eye disease: Proceedings of the OCEAN group meeting. Ocul. Surf..

[49] Messmer E.M. (2022). Pathophysiology of dry eye disease and novel therapeutic targets. Exp. Eye Res..

[50] Craig J.P., Nichols K.K., Akpek E.K., Caffery B., Dua H.S., Joo C.K., Liu Z., Nelson J.D., Nichols J.J., Tsubota K. (2017). TFOS DEWS II Definition and Classification Report. Ocul. Surf..

[51] Roda M., Corazza I., Bacchi Reggiani M.L., Pellegrini M., Taroni L., Giannaccare G., Versura P. (2020). Dry Eye Disease and Tear Cytokine Levels—A Meta-Analysis. Int. J. Mol. Sci..

[52] Garriz A., Morokuma J., Bowman M., Pagni S., Zoukhri D. (2022). Effects of Proinflammatory Cytokines on Lacrimal Gland Myoepithelial Cells Contraction. Front. Ophthalmol..

[53] Li J.M., Lu R., Zhang Y., Lin J., Hua X., Pflugfelder S.C., Li D.Q. (2021). IL-36α/IL-36RA/IL-38 signaling mediates inflammation and barrier disruption in human corneal epithelial cells under hyperosmotic stress. Ocul. Surf..

[54] Li L., Jin R., Li Y., Nho J., Choi W., Ji Y., Yoon H., Yoon K. (2020). Effects of Eurya japonica extracts on human corneal epithelial cells and experimental dry eye. Exp. Ther. Med..

[55] Seen S., Tong L. (2018). Dry eye disease and oxidative stress. Acta Ophthalmol..

[56] Fernandez-Torres M.A., Lledó V.E., Perez de Lara M.J., Guzman-Aranguez A. (2022). Effects of hyperosmolarity on annexin A1 on ocular surface epithelium in vitro. Exp. Eye Res..

[57] Nakamura S., Shibuya M., Nakashima H., Hisamura R., Masuda N., Imagawa T., Uehara M., Tsubota K. (2007). Involvement of oxidative stress on corneal epithelial alterations in a blink-suppressed dry eye. Investig. Ophthalmol. Vis. Sci..

[58] Wakamatsu T.H., Dogru M., Tsubota K. (2008). Tearful relations: Oxidative stress, inflammation and eye diseases. Arq. Bras. Oftalmol..

[59] Saccà S., Cutolo C., Ferrari D., Corazza P., Traverso C. (2018). The Eye, Oxidative Damage and Polyunsaturated Fatty Acids. Nutrients.

[60] Chen K., Li Y., Zhang X., Ullah R., Tong J., Shen Y. (2022). The role of the PI3K/AKT signalling pathway in the corneal epithelium: Recent updates. Cell Death Dis..

[61] Hafez A.A., Jamali Z., Khezri S., Salimi A. (2021). Thymoquinone reduces mitochondrial damage and death of cardiomyocytes induced by clozapine. Naunyn-Schmiedeberg’s Arch. Pharmacol..

[62] Farkhondeh T., Samarghandian S., Borji A. (2017). An overview on cardioprotective and anti-diabetic effects of thymoquinone. Asian Pac. J. Trop. Med..

[63] Dur A., Kose H., Kocyigit A., Kocaman O., Ismayilova M., Sonmez F.C. (2016). The anti-inflammatory and antioxidant effects of thymoquinone on ceruleine induced acute pancreatitis in rats. Bratisl. Lek. Listy.

[64] Liang J., Lian L., Wang X., Li L. (2021). Thymoquinone, extract from Nigella sativa seeds, protects human skin keratinocytes against UVA-irradiated oxidative stress, inflammation and mitochondrial dysfunction. Mol. Immunol..

[65] Almalik A., Alradwan I., Majrashi M.A., Alsaffar B.A., Algarni A.T., Alsuabeyl M.S., Alrabiah H., Tirelli N., Alhasan A.H. (2018). Cellular responses of hyaluronic acid-coated chitosan nanoparticles. Toxicol. Res. (Camb).

[66] Ke C., Sun L., Qiao D., Wang D., Zeng X. (2011). Antioxidant acitivity of low molecular weight hyaluronic acid. Food Chem. Toxicol..

[67] Lin J., Wu H., Wang Y., Lin J., Chen Q., Zhu X. (2016). Preparation and ocular pharmacokinetics of hyaluronan acid-modified mucoadhesive liposomes. Drug Deliv..

[68] Zheng Q., Li L., Liu M., Huang B., Zhang N., Mehmood R., Nan K., Li Q., Chen W., Lin S. (2020). In situ scavenging of mitochondrial ROS by anti-oxidative MitoQ/hyaluronic acid nanoparticles for environment-induced dry eye disease therapy. Chem. Eng. J..

[69] Huerta-Angeles G., Němcová M., Příkopová E., Šmejkalová D., Pravda M., Kučera L., Velebný V. (2012). Reductive alkylation of hyaluronic acid for the synthesis of biocompatible hydrogels by click chemistry. Carbohydr. Polym..

